# Policy innovation of construction waste management in Xi’an, China: A SWOT analysis of incentive mechanisms

**DOI:** 10.1371/journal.pone.0339386

**Published:** 2025-12-18

**Authors:** Han Liu, Siqing Yang, Muhammad Noor Hisyam Jusoh

**Affiliations:** 1 School of Economics and Management, Ankang University, Ankang, China; 2 Faculty of Engineering Technology and Built Environment, UCSI University, Cheras, Kuala Lumpur, Malaysia; Xi'an University of Architecture and Technology, CHINA

## Abstract

This study uses Xi’an as a case to explore incentive mechanisms and optimization pathways in construction waste management. Drawing on policy text analysis, semi-structured interviews, and the Delphi method, and employing the SWOT-TOWS framework, the research systematically evaluates the strengths, weaknesses, opportunities, and threats of current policies. The findings indicate that while Xi’an benefits from fiscal support and standard-setting, inefficiencies in policy enforcement and low market trust continue to hinder effective resource utilization. To address these challenges, the study proposes a phased strategy: in the short term, improve regulatory closure and enforcement mechanisms; in the medium term, strengthen capacity building, establish market-supporting measures, and stabilize demand; and in the long term, promote institutionalization, financial diversification, and large-scale development. Such measures aim to facilitate the transition from “policy-driven pilot projects” to “market-driven normalization.” This study provides practical insights for Xi’an and other cities at a similar stage of development in building more comprehensive policy support frameworks for sustainable construction waste management.

## 1. Introduction

In most developing countries, socioeconomic development is closely intertwined with construction activity, and China is no exception. Over the past three decades, the country has witnessed rapid progress in urbanization and public infrastructure development, accompanied by an unprecedented expansion of the construction industry [1]. However, this rapid growth has also posed multiple challenges to the environment and natural resources. In 2024, Construction Waste (CW) in China reached 2.41 billion tons, accounting for 25.9% of municipal solid waste, indicating that it has become one of the dominant components [2]. CW generally arises from new construction, renovation, and demolition activities involving buildings, roads, bridges etc [3]. Typical waste materials include discarded aggregates, bricks, reinforcing steel, plastic packaging, timber, and similar by-products [4].

At present, China’s Construction Waste Management (CWM) remains at a relatively early stage. A large proportion of CW is still not properly treated after generation, thereby exacerbating its adverse impacts [5]. The role of the government is considered crucial for solving these issues [6]. In recent years, government agencies have actively introduced a range of policies and regulations aimed at mitigating the negative effects of CW, such as imposing stricter controls on illegal dumping and promoting higher rates of waste material reuse. While these measures have achieved some progress, the overall impact remains limited [7]. Therefore, exploring policy innovations to improve the effectiveness of CWM is both necessary and meaningful.

In recent years, international studies have examined various incentive mechanisms for promoting CWM, including market-based instruments (e.g., landfill taxes, recycling credits), regulatory measures, and public-private collaboration frameworks [8–10]. These studies, however, largely focus on mature governance environments in better economies, where institutional capacity and policy enforcement are relatively strong. In contrast, emerging economies such as China face distinctive challenges, including fragmented policy implementation and limited market maturity. Xi’an, as a rapidly urbanizing city in Western China and a core node of the One Belt One Road (OBOR), provides a representative case for examining how incentive mechanisms are localized and adapted under transitional governance conditions. Therefore, this study situates the Xi’an case within the broader international discourse on sustainable CW governance to bridge global insights with local policy practice.

Over the past two decades, Xi’an has undergone rapid urbanization, with annual CW generation significantly exceeding the provincial average [11]. As announced by Shaanxi government, Xi’an produced 103.96 million tons of CW, much more than the output of other cities in the province such as Xianyang (4.63 million tons) and Ankang (4.85 million tons) [12]. Furthermore, Xi’an was designated by the Ministry of Housing and Urban-Rural Development as a national pilot city for CWM in 2018. Since then, the city has introduced and implemented a series of policies and mechanisms that ranging from subsidies and clearance procedures to the active involvement of private capital. By 2024, these measures had raised the resource utilization efficiency of CW in Xi’an to 53%, well above the national average [12]. In this context, examining the incentive mechanisms embedded in Xi’an’s CWM policies offers valuable reference for cities of similar economic level, urban scale, and waste generation, while also providing useful insights for policy development at the national level.

In China, municipal governments have adopted varied strategies to promote CW reduction and recycling. For instance, Shenzhen and Suzhou have introduced market-based measures such as deposit-refund systems and differential disposal fees to incentivize on-site waste segregation, while Beijing has implemented stricter enforcement frameworks emphasizing mandatory recycling quotas and traceability systems for construction debris [13–15]. In contrast, Xi’an’s approach remains primarily administrative and relies heavily on government-led directives with limited fiscal incentives and enterprise participation. This divergence highlights an important policy innovation gap: while developed cities have gradually shifted toward hybrid governance models integrating market and regulatory tools, western regions like Xi’an are still in the early stages of policy experimentation. Current CWM literature has paid insufficient attention to these regional differences, especially regarding how incentive structures operate under different economic and institutional contexts. This study therefore addresses this gap by systematically analysing Xi’an’s incentive policies and evaluating their implementation dynamics from a multi-stakeholder perspective.

Unlike previous studies that applied SWOT merely as a descriptive diagnostic tool, this research integrates the Delphi validation approach to enhance the objectivity and reliability of SWOT factor identification. The Delphi-assisted SWOT not only reduces subjectivity inherent in traditional qualitative assessments but also facilitates a more rigorous synthesis of multi-stakeholder insights from government, contractors, and recycling enterprises [16]. Furthermore, by focusing on the incentive mechanisms at the municipal level, this study extends existing theories of policy implementation in CWM by uncovering how fiscal and regulatory incentives interact in local governance contexts such as Xi’an. Accordingly, this study employs a TOWS-based matrix framework to explore policy optimization pathways in the case of Xi’an. This provides a novel analytical lens for understanding localized incentive structures in developing-country settings.

## 2. Methodology

### 2.1. Research design

To explore the applicability and optimization pathways of policy incentive mechanisms in CWM, this study adopts a qualitative research approach. Specifically, it draws on policy document analysis, semi-structured interviews, and SWOT analysis, with Xi’an selected as the case study site. The aim is to gain a multidimensional understanding of the intrinsic connections among policy formulation, implementation, and subsequent feedback.

**Fig 1:**
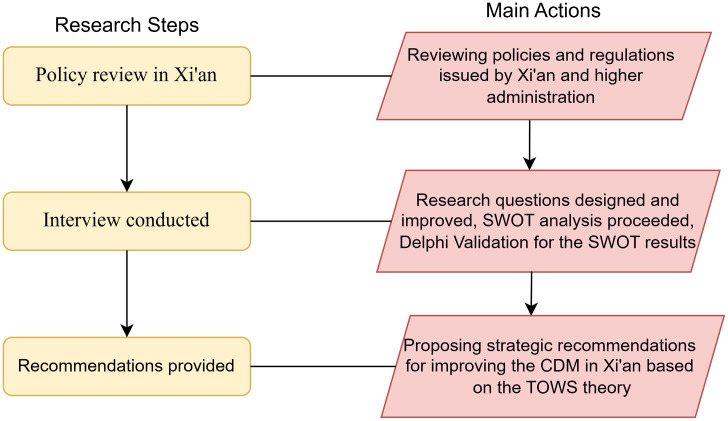
Three steps and main actions of the research.

The study proceeds through three main steps, as illustrated in Fig 1. First, a literature review was conducted to collect and examine relevant policy documents issued by the Xi’an municipal government and the provincial government since 2015, with particular attention to policies promoting incentives for waste management during this period. To facilitate subsequent comparison within the SWOT analysis, the policy review was structured around several key dimensions, including types of policy instruments, scope of application, implementation mechanisms, and target groups. Second, to address the limitations of relying solely on policy texts and to better capture their practical effects, semi-structured interviews were conducted. Finally, by integrating the key findings from both the policy documents and the interviews, the study employed a SWOT analytical framework.

### 2.2. Data sources and sampling

The data for this study were drawn from two main sources. The first source consists of a review of literature related to incentive policies for CWM, including primary policy documents, government guidelines, and industry reports. At the administrative level, in addition to official documents issued by the Xi’an municipal government, guiding policies from higher-level authorities—such as the Ministry of Housing and Urban-Rural Development (MOHURD) and the Ministry of Ecology and Environment (MEE)-were also examined. Since these higher-level policies are closely linked to municipal level policymaking, their inclusion ensures the systematic nature and comprehensiveness of the dataset, thereby providing a solid foundation for analyzing policy incentive mechanisms [17].

The second source comprises semi-structured online interviews with nine selected respondents. The interview framework was organized around the four dimensions of SWOT (strengths, weaknesses, opportunities, and threats) to investigate the practical effects of existing incentive policies in Xi’an, the difficulties in their implementation, the responses of stakeholders, and recommendations for improvement.

Careful selection of interview participants was crucial to the research design. As noted in previous studies, CWM policies are most directly shaped by three key actors: government authorities, construction enterprises, and recycling organizations [18]. A balanced exploration of policymaking, application, and feedback across these three groups is therefore essential to improving policy effectiveness [19]. Furthermore, previous studies using empirical data reached saturation within a narrow range of interviews (9–17) [20]. Therefore, this study adopted purposive sampling to ensure representation of key stakeholder groups directly involved in CWM in Xi’an. Nine participants were selected, comprising three government officials (G1-G3), three representatives from construction enterprises (C1-C3), and three from recycling units (R1-R3). Selection criteria included at least ten years of experience in policy implementation, project management, or recycling operations, and direct involvement in local CWM practices. Participants were recruited through professional contacts and departmental referrals within Xi’an’s housing and urban development online system. This approach ensured the inclusion of individuals with firsthand knowledge of policy execution and industry challenges.

This study involved human participants. Ethical approval was granted by UCSI University, Malaysia (Reference code: IEC-2024-FETBE-0002). All interviews were conducted online and lasted between 30 and 60 minutes, from March 1st to April 20th, 2025. Informed consent was obtained from all participants prior to their involvement in the study. Written consent was obtained, and participants were provided with both written and verbal explanations of the study’s purpose, procedures, potential risks, and their rights to withdraw at any time without consequence.

### 2.3. Interview question design

The interview design was structured around four dimensions, with each dimension containing two to three guiding questions, thereby reflecting the practical implementation of incentive policies in Xi’an. The formulation of these questions was also informed by insights from prior related studies [21]. The first set of questions focused on identifying the achievements and positive outcomes of current policies in practice. The second explored problems and difficulties embedded in policy design or arising during implementation. The third examined external conditions and emerging trends that may facilitate policy improvement and execution. The fourth investigated external factors that could hinder policy implementation or diminish its effectiveness. The full list of questions is provided in Appendix A.

During the interviews, the content was slightly adjusted according to the role of each participant. For instance, government representatives emphasized institutional dimensions, while construction enterprises and recycling organizations concentrated more on implementation issues. Each interview was audio-recorded, transcribed, and coded. Using a combination of open coding and thematic categorization, common viewpoints were extracted to inform the subsequent matrix and strategy analyses.

### 2.4. Data analysis

Following the collection of policy documents and interview data, all raw materials were systematically coded and thematically categorized. Interview transcripts were subjected to open coding, with responses classified using an Excel-based framework under the four SWOT dimensions. Representative viewpoints under each dimension were consolidated into key entries and repeatedly cross-checked to ensure consistency in coding. To enhance coding reliability, two researchers independently coded the transcripts and then compared and discussed discrepancies until full agreement was reached. Once coding was completed, the data were organized into a preliminary SWOT matrix, structured along internal and external factors. As noted by Li, Davies [22], the validity and authority of SWOT findings can be enhanced by employing the Delphi method.

Drawing on the Delphi method, four experts with backgrounds in CWM or related policy research in Xi’an were invited to review the initial SWOT draft via email. This sample size aligns with methodological guidance suggesting that small, expert-focused panels (3–5 participants) are appropriate for specialized policy domains where expert consensus, rather than population representativeness, is the primary goal [23]. The selection followed two criteria: (1) a minimum of ten years of professional experience in CWM or related policy implementation; and (2) active involvement in policy-making, regulation, or project execution within Xi’an or Shaanxi Province. The Delphi process was conducted in two iterative rounds. In the first round, participants reviewed and rated the relevance and accuracy of the coded SWOT elements derived from the interview analysis. In the second round, they were asked to reconsider their ratings in light of the group’s summarized feedback. Consensus was defined as achieving a Kendall’s coefficient of concordance (W) ≥ 0.70 or a 75% agreement rate among experts on the categorization and priority ranking of each element [24]. Items not meeting this threshold were revised or merged through discussion and re-evaluation until the threshold was reached. This process ensured reliability and convergence in the interpretation of qualitative data.

Building on this foundation, the study proceeded to strategy development using the TOWS matrix method, including SO, ST, WO and WT. As highlighted by Auer and Rauch [25], this approach allows for systematic decision-making by cross-matching external opportunities and threats with internal strengths and weaknesses. These strategies provide actionable recommendations for optimizing local incentive policies.

## 3. Results and discussion

### 3.1. Policy text analysis results

#### 3.1.1. Current policy of CWM in Xi’an.

This section is based on a systematic review of municipal documents issued in Xi’an between 2015 and 2025, relevant provincial-level policies from Shaanxi, and national guidelines and standards on CW and resource utilization issued by central ministries. Using content coding, the “incentive clauses” were categorized according to type, target groups, and implementation status, thereby laying the foundation for subsequent cross-validation with interview evidence and SWOT mapping. The detailed results are presented in Table 1, which includes not only the policies in Xi’an but also higher-level regulations directly relevant to the city.

**Table 1:** Main policies implemented in Xi’an.

Code	Year	Laws and Regulations	Main content
P1	2015	National Industry Standard “Recycled Coarse Aggregate for Concrete	Standardizes terminology, classification, quality indicators, and testing methods for recycled aggregates
P2	2017	Construction Waste Management Regulations in Xi’an	Clarifies responsibilities for full-process management of CW, reporting and disposal requirements; defines administrative duties and penalty measures
P3	2018	National Financial Support and Demonstration Project Channels for Promoting Construction Waste Reduction and Resource Utilization	Provides central matching funds and technical promotion funding for certified demonstration resource utilization projects
P4	2019	Implementation Opinions on Promoting Construction Waste Resource Utilization in Shaanxi Province	Provincial pilot funding, promotion of technical standards, and supporting measures for municipal pilot projects
P5	2019	Local Standards for Recycled Aggregates at Shaanxi Province	Issues technical specifications for applications such as recycled aggregate concrete and recycled aggregate pumping, clarifying quality requirements
P6	2020	Guiding Opinions on Promoting Construction Waste Reduction	Emphasizes source reduction, classified collection, resource utilization, and pilot promotion, proposing quantitative targets and technical pathways
P7	2020	Implementation Measures for Strengthening Construction Waste Resource Utilization in Xi’an	Clarifies recycled aggregate qualifications, planning requirements for resource utilization sites, financial subsidies, and cancellation procedures (partial pilot)
P8	2022	Urban Renewal Measures in Xi’an	Defines approval processes and green channels for urban renewal projects, providing support and convenience for projects using green/recycled materials
P9	2023	Implementation Plan for Green Building and Prefabricated Building Development in Xi’an	Offers specialized support and incentives for projects using recycled materials and prefabricated construction technologies

The findings indicate that over the past decade, CWM policies have undergone notable evolution. From 2015 to 2018, national and provincial regulations, along with industry standards, were gradually refined, establishing the institutional and regulatory groundwork for quality control and supervision (see P1 and P5). Between 2017 and 2021, Xi’an introduced pilot programs and detailed implementation measures, with local policy documents explicitly defining requirements for recycled materials, their applications, and pilot subsidy mechanisms (see P2, P4, and P7). During 2022 and 2023, the policy orientation shifted from macro-level frameworks toward more practical operational guidelines, including support for urban renewal and housing renovation, incentives for prefabricated buildings, detailed green subsidy measures, and streamlined approval processes (see P8 and P9). Meanwhile, external opportunities—such as fiscal support and demonstration project channels from higher-level authorities, which provided complementary resources for local implementation (see P3 and P6).

#### 3.1.2. Comprehension policy framework.

Based on the review of current policy content in Xi’an, the policy framework can be summarized into three main dimensions:

(1) Direct policy incentives.

Documents P2 and P7 explicitly demonstrate that Xi’an has established mechanisms for financial subsidies and write-offs (e.g., production-based subsidies, tax reductions, or one-time rewards for enterprises using recycled construction materials). As noted by Liu and Teng [26], such economic incentives are the most effective in the short term, as they directly reduce costs for recycling enterprises and increase their willingness to participate. However, analysis of the guidance issued by the Shaanxi Provincial Department of Ecology and Environment indicates that the timeliness and complexity of subsidy disbursement significantly affect firms’ cash flow and sustained commitment. In other words, the difference between “having subsidies” and “having subsidies delivered promptly” is critical to determining their actual motivational effect.

(2) Enhancement of standards.

Policies P1 and P5 emphasize the establishment of technical standards and regulatory norms. For example, standards for recycled aggregates provide strong institutional support for the marketization of recycled materials. In a study on the development of the CW recycling market, argued that clear quality benchmarks enhance buyer confidence and facilitate the entry of recycled materials into higher-end engineering projects [27]. At the same time, small and medium-sized enterprises (SMEs) may face pressure in meeting these higher standards. As identified in field investigation by Bao, Lu [5], stricter performance requirements increase the costs of product testing, technological upgrades, and certification, effectively raising market entry barriers for SMEs. In the context of SWOT analysis, this duality can be interpreted as both a strength (institutional guarantee) and a weakness (compliance costs). Strategies for addressing these issues will later consider the potential role of financial and technical support.

(3) Incentives in specific construction contexts.

Policies P8 and P9 highlight the government’s use of targeted incentives within specific construction scenarios. These align with national-level macro policies in the real estate sector. For instance, in 2020, MOHURD emphasized the importance of urban renewal and old housing renovation to replace indiscriminate demolition and uncontrolled urban expansion. In this context, P8 provides guidance for urban regeneration and renovation projects, while P9 focuses on green buildings. Both policies feature mechanisms such as tiered subsidies and streamlined approval processes. Incentives designed for specific contexts are more likely to achieve stronger effects and can serve as pilot projects. However, their effectiveness remains dependent on the scope of pilot programs and the scale of fiscal support. As highlighted by Wang, Jiang [6], policy measures from leading cities are often difficult to replicate nationwide. For Xi’an, its dual role as both a provincial capital and a designated pilot city for CWM creates opportunities to secure more direct and substantial financial support.

#### 3.1.3. Preliminary policy-to-SWOT mapping.

Based on the above content coding and interpretation, a preliminary understanding of the incentive policies for CWM in Xi’an has been established. These findings allow for the initial mapping of key policy points into candidate SWOT items. As shown in Table 2, the results are divided into two parts: primary mappings and secondary mappings. The primary mappings are categorized according to policy level, distinguishing between local and higher-level policies. Following the approach suggested in Teoli, Sanvictores [28] SWOT study, conditions directly relevant to local implementation are treated as strengths (S), while conditions indirectly supportive of policy outcomes are classified as opportunities (O). This framework is also adopted in the present study. In addition, it is recognized that certain policies may generate both positive and negative effects during implementation. Accordingly, the Table also presents secondary mappings that capture these dual impacts, providing a more nuanced basis for the subsequent SWOT analysis.

**Table 2:** Preliminary mapping results of incentive policies to SWOT analysis in Xi’an.

Code	Policy Highlights	Primary Mapping	Secondary Mapping	Key reasons
P1	Industry Standards for Recycled Aggregates at National Aspect	S	O/W	National standards provide overarching support and cross-regional acceptance (strength/opportunity); local adaptation and cost issues may pose weaknesses.
P2	Municipal Regulations: Full-process management, responsibilities, and penalties (including incentive clauses)	S	W/O	Regulations provide legal and institutional foundations (strength); incomplete supporting measures or weak enforcement may become weaknesses; regulations can help secure higher-level support (opportunity).
P3	Central Demonstration Projects and Fiscal Support Channels	O	S/T	Central funding/demonstration projects represent significant external opportunities; approval enhances demonstration effects (strength); however, high application thresholds may pose threats if support is not obtained.
P4	Provincial Implementation Opinions: Provincial Funding and Pilot Support	O	W	Provincial support is an external opportunity; inadequate fund allocation or mismatched execution may lead to implementation gaps (weakness).
P5	Local Technical and Quality Standards for Recycled Aggregates (Local Regulations)	S	W/O	Standards enhance market trust and quality assurance (strength); however, compliance costs and insufficient testing capacity may form weaknesses; standardization also facilitates promotion (opportunity).
P6	Ministerial Guidance: Source reduction and resource utilization promotion	O	S	Higher-level guidance creates external opportunities for local support and demonstration projects; also provides a foundation for local institutional design (strength).
P7	Municipal Implementation Rules: Subsidy Standards and Reimbursement Procedures	S	W/T	Subsidies serve as direct incentives in policy text (strength); however, delayed reimbursement processes or slow disbursement may weaken effectiveness (weakness); long-term inefficacy or loss of confidence poses threats.
P8	Urban Renewal Measures: Green Channels and Project Subsidies for Redevelopment	S	O/W	Scenario-specific incentives for redevelopment directly align with demolition contexts (strength); create opportunities for demonstration; limited pilot scope or insufficient funding may become weaknesses.
P9	Green/Prefabricated Building Plans: Incentives for Using Recycled Materials	S	O/W	Stimulates demand and boosts market growth (strength); synergies with green policies present opportunities; limited coverage or inconsistent standards may form weaknesses.

Drawing on Table 2, the policy texts can be preliminarily classified and discussed as follows. Policies P2, P4, and P7 constitute the institutional foundation for current municipal-level financial incentives. At the textual level, such quantified subsidies directly enhance the short-term economic motivation of recycling enterprises and are therefore mapped as a strength. However, as indicated in the table and initial observations, delays in disbursement and the complexity of reimbursement procedures remain contested issues. These factors would be further examined in Section 3.2 through interview evidence to assess whether they undermine the intended policy effects-potentially reclassifying them as a weakness. Policies P1 and P5, which introduce provincial and national standards for recycled aggregates, provide higher-level institutional guarantees that facilitate market entry for recycled materials. This is reflected in the textual analysis as a strength. Yet, the standardization process also entails additional testing and technological upgrading costs that may suppress the participation of smaller recycling firms in the short term, suggesting a potential weakness. Policies P3, P8, and P9 focus on urban renewal, old housing renovation, and central demonstration project channels, forming a combined mechanism of incentives of specific urban renewal projects and demonstration effects. The texts suggest that such measures can directly stimulate recycling behavior in demolition-intensive areas (opportunity) while amplifying successful practices through demonstration projects (strength). Nonetheless, their overall effectiveness depends on the scale of pilot coverage and fiscal support. If financial resources or implementation reach are limited, the demonstration effect may struggle to diffuse more widely.

Overall, the policy framework can be summarized into three categories. The first consists of policies that directly embody incentive instruments, specifying concrete measures and subsidy levels. The second includes higher-level or supporting documents, which primarily function as external opportunities providing a broader policy context. The third comprises technical and standard-setting documents, whose effects are twofold—serving as institutional safeguards while potentially increasing compliance costs. Based on this preliminary categorization and textual assessment, the next section presents semi-structured interviews with nine respondents. By examining their perceptions and evaluations of the above policy elements, and drawing on their responses to the four thematic dimensions of the interview questions, the study further validates how policies operate in practice and highlights the gaps between policy texts and their implementation.

### 3.2. Validation of policy texts through semi-structured interviews

#### 3.2.1. Main interview results content.

Semi-structured interviews were conducted with nine respondents, and the transcripts were subsequently coded and analyzed. Based on the four categories of guiding questions, participants’ responses and corresponding viewpoints were summarized. The results in Table 3 reveal two main trajectories from the interview evidence. On the one hand, most respondents affirmed the short-term effectiveness of incentives (e.g., urban renewal pilots) and output-based subsidies, with 7 out of 9 participants highlighting their demonstrative impact in pilot projects. On the other hand, feedback was more concentrated and consistent regarding implementation challenges. In particular, delays in subsidy reimbursement or disbursement were unanimously recognized as a problem (9/9), while high compliance and testing costs, along with insufficient cross-departmental coordination, were repeatedly mentioned by the majority of respondents. External opportunities and threats were also identified, most notably the supportive role of higher-level regulations versus challenges such as weak local enforcement and market instability, which received broad agreement among participants. Overall, the findings suggest that while Xi’an’s current incentive instruments are comprehensive in design, systemic bottlenecks persist in the latter stages of the “design–implementation” chain. The high-frequency items indicate the relative priority of these bottlenecks for future policy adjustment.

**Table 3:** The main results summarized from the interview.

Question set	Main questions	Number of Respondents Supporting/ Agreeing (n = 9)	Key Insights
Effectiveness	Which incentives are most effective? Are there successful cases? Has enforcement improved?	7/9 identified incentives linked specific urban renewal projects (e.g., urban renewal) and volume-based subsidies as the most effective in the short term; 6/9 reported localized demonstration cases.	Incentives combined with subsidies show effects in pilot zones, but slow subsidy disbursement undermines sustainability
Implementation Barriers	What are the main shortcomings? Do policies deviate from practice? How is interdepartmental coordination?	9/9 pointed to slow subsidy verification/disbursement; 7/9 cited high testing/compliance costs; 6/9 noted poor interdepartmental coordination	Procedural complexity, capacity constraints, and governance frictions are the main causes
External Opportunities	What technological/market opportunities exist? Any role for public/green demand?	8/9 highlighted green finance, carbon markets, and long-term government procurement as key opportunities; 6/9 were optimistic about mobile crushing and online monitoring technologies	Technology and green finance could transform short-term subsidies into long-term incentives
External Threats	Are there risks from illegal disposal, regulatory gaps, or market limitations?	7/9 noted that illegal dumping and low-cost noncompliance remain problems; most expressed concerns about fiscal instability	Illegal disposal, market uncertainty, and short-termism in policy are the primary threats

The distributed evidence in the Table 3 can be interpreted from two perspectives: policy execution and supply-demand dynamics. From the execution side, the slow disbursement of subsidies and lengthy approval procedures suggest that the timeliness and accessibility of policies are constrained. In public policy implementation theory, this phenomenon is often described as incentive erosion, whereby policies formally exist but their intended effects are weakened by obstacles in the execution process [29]. From the supply-demand side, while stricter quality standards have enhanced market confidence, the absence of sufficient public testing capacity and technical support has produced a “threshold effect”, temporarily crowding out the participation of small- and medium-sized recycling enterprises. As noted in the literature that policy evaluation should therefore go beyond assessing the mere “existence” of policies and place greater emphasis on implementation efficiency, capacity alignment, and institutional coordination as key dimensions of analysis [30].

#### 3.2.2. Discussion based on the SWOT.

Next, the interview results were synthesized into a SWOT framework, as shown in Table 4. The coding followed an open-axial-selective procedure, grouping recurrent concepts into four major categories: strengths, weaknesses, opportunities, and threats. Each code represents a cluster of interview statements and then will achieve expert consensus in two Delphi rounds. And In this section, the four dimensions then been discussed in detail to provide a clearer understanding of the current policy landscape in Xi’an and to inform directions for future improvement.

**Table 4:** SWOT Elements of the policy evaluation in Xi’an.

Category	Element	Quotations
Strengths (S)	S1. Incentives linked to specific urban renewal projects and municipal subsidies show short-term effectiveness in pilot zones	G1: “The municipal incentive policy worked quite well in pilot renewal areas—contractors were visibly more proactive.” C2: “In the pilot zone, our team received subsidies on time, which encouraged better waste sorting and documentation.” R1: “Once subsidies were announced, recycling plants increased their operating shifts to meet the rising demand.”
	S2. Quality standards and higher-level policy guidance enhance market confidence	G2: “After provincial technical standards were released, developers became more willing to use recycled aggregates.” R2: “Having clear guidelines gives us confidence to defend product quality during tender evaluations.” C1: “Before the standards, everyone doubted the safety of recycled materials—now we finally have a common reference.”
	S3. Central/provincial demonstration funds provide resource opportunities for local governments	G3: “The demonstration fund allowed us to initiate model projects and show measurable results to the public.” R3: “Without the provincial support fund, purchasing crushing equipment would have been impossible for us.” C2: “These funds helped local governments build partnerships between contractors and recyclers.”
Weaknesses (W)	W1. Slow subsidy disbursement/verification and cumbersome procedures	C3: “After project completion, we waited nearly half a year for the subsidy approval.” R2: “The verification involves too many forms and departments; it delays our cash flow.” G2: “Our internal coordination for subsidy release is still inefficient—we know this is a problem.”
	W2. Insufficient testing capacity and high compliance costs squeeze out SMEs	R3: “Testing every batch costs too much; small firms like ours can hardly survive under current rules.” G1: “The city has only a few certified labs, and the queue for testing recycled aggregates is very long.” C1: “The compliance cost is high, and only large contractors can afford frequent testing.”
	W3. Weak interdepartmental coordination and lack of information sharing	G2: “Different bureaus hold their own data systems, and cross-departmental sharing is still limited.” C3: “We often submit the same information separately to the construction and environmental departments.” R1: “Without a unified data platform, enforcement and reporting are both inefficient.”
Opportunities (O)	O1. Green finance, carbon markets, and long-term government procurement open market-oriented pathways	G3: “Green bonds and carbon trading could provide sustainable funding channels for recycling enterprises.” R2: “If government procurement gives preference to recycled materials, market confidence will grow quickly.” C2: “Carbon credit mechanisms could make recycled materials financially more competitive.”
	O2. Large-scale urban renewal provides stable demand scenarios for resource utilization	G1: “Xi’an’s old city renewal ensures a long-term and predictable flow of CW for recycling.” C1: “These projects give us stable contracts, unlike short demolition jobs.” R3: “Urban renewal keeps our recycling plant operating continuously without worrying about raw material supply.”
	O3. Mobile crushing, online monitoring, and digital regulation reduce costs and improve enforcement	R1: “Mobile crushing equipment saves transportation cost and minimizes illegal dumping.” C2: “Digital monitoring systems let us trace every truckload and avoid penalties.” G2: “Smart regulation reduces manual inspection work and increases transparency.”
Threats (T)	T1. Illegal dumping and enforcement difficulties distort market competition	G3: “Despite higher penalties, illegal dumping still occurs in suburban areas.” C3: “Some competitors avoid waste fees by dumping at night, which undermines fair competition.” R1: “Weak enforcement lets irresponsible players undercut those who comply with rules.”
	T2. Weak market demand and low price competitiveness of recycled materials	C1: “Developers often reject recycled aggregates because they still doubt the quality.” R2: “Recycled products are cheaper, but clients don’t see enough value to justify the switch.” G2: “Market recognition is still low; awareness campaigns haven’t reached private developers.”
	T3. Fiscal subsidies are temporary and vulnerable to short-term policy risks	R3: “We hesitate to invest heavily because the subsidy policies may change each year.” C2: “Without long-term guarantees, companies can’t plan sustainable recycling capacity.” G1: “The subsidy system depends on annual budgets, which makes it difficult to maintain continuity.”

(1) Discussion on the strength

Xi’an’s most notable policy strength lies in the combined mechanism of incentives on specific urban renewal projects and fiscal subsidies. In high-density demolition contexts such as old city renovation, this combination has proven effective in accelerating the adoption of recycled materials (corresponding policies: P2 P7 P8 P9). Interview data indicate that construction firms show significantly higher willingness to adopt recycled materials in designated demonstration zones. Government support-through streamlined approval channels and one-off project subsidies—has reduced upfront transaction costs, generating observable demonstration effects. This aligns with the classic logic of the “demonstration–replication” pathway in policy diffusion theory, where short-term fiscal incentives and administrative facilitation jointly foster replicable business models [31]. Based on this, it is recommended that successful urban renewal pilots be consolidated into “replication packages” (combining fiscal, administrative, and technical service components) and scaled up through applications to P3 (central demonstration funds), thereby transforming S1 into a city-wide institutional capacity.

The establishment of quality standards (P1/P5) provides an institutional safeguard for recycled construction materials entering public works projects. Both experts and recycling enterprises confirmed that these standards enhance purchaser confidence. While the positive effect lies in reducing information asymmetry, without adequate testing capacity and certification services, standards risk becoming restrictive thresholds [32]. To ensure the effective realization of S2, standard development should proceed in parallel with the expansion of public testing and certification capacity, potentially supported by municipal funding or provincial-level matching funds (P4).

Finally, central and provincial demonstration funding channels (P3) provide external support by supplying financial resources, introducing advanced technologies, and amplifying demonstration effects. Respondents generally agreed that demonstration funds exert a “leveraging effect,” as approved projects are more likely to attract private investment and corporate technological input. However, the competitive and one-off nature of such funds must be acknowledged. In practice, policy effectiveness would be enhanced by coupling funding with application support and follow-up mechanisms, ensuring that demonstration projects evolve into sustained drivers of change rather than isolated events [33].

(2) Discussion on the weakness

Respondents exhibited a high degree of consensus regarding the issue of subsidy disbursement efficiency (W1). Although municipal policy documents (P2, P7) clearly stipulate the subsidy standards and verification procedures, in practice the allocation cycle is lengthy, the documentation requirements are cumbersome, and multiple departmental reviews are involved. As a result, enterprises are often placed under financial pressure due to a “pay first, reimburse later” mechanism. According to Manheim [34], this reflects a structural problem of “insufficient immediacy of incentives,” whereby the timeliness of economic incentives determines their marginal stimulating effect. Consequently, some scholars argue that without reforms to the disbursement mechanism-such as piloting advance payments, adopting a credit-based approach, or implementing performance-linked installment disbursements-fiscal subsidies are unlikely to achieve their intended policy outcomes [35].

The issues of insufficient testing capacity and high compliance costs (W2) were repeatedly emphasized by respondents (corresponding to P1/P5). In the absence of public testing platforms and subsidies for technological upgrading, national and provincial standards risk forcing small and medium-sized recycling enterprises out of the market, as they are unable to bear the costs of testing and facility modification. This, in turn, reduces supply elasticity and weakens the diversity of regional supply. Another systemic weakness highlighted is the problem of cross-departmental coordination (W3), particularly concerning the division of responsibilities and implementation guidelines in P2 and P4. Interviews revealed that duplicated approval procedures, lack of mutual recognition of information, and ambiguous lines of accountability increase both project delays and uncertain costs. As Aaltonen and Turkulainen [36] argues, addressing these issues requires institutionalized solutions: administrative process reengineering, the establishment of data-sharing platforms, and the introduction of clear SLAs (service-level agreements) and responsibility matrices would substantially improve administrative efficiency.

(3) Discussion on the opportunity

In the interviews, respondents expressed strong expectations regarding green finance and carbon markets (O1), identifying them as a critical pathway to transform one-off fiscal subsidies into long-term, market-oriented incentives (P3 and P9). From an institutional innovation perspective, integrating recycled materials into green certification schemes, green credit products, and carbon credit systems could provide enterprises with sustained financing advantages and alternative revenue streams [37]. The large-scale advancement of urban renewal projects (O2) offers a stable demand scenario and represents an opportunity to scale up pilot initiatives (P8). Respondents emphasized that urban renewal contexts inherently possess the advantage of spatial proximity between producers and end-users. This aligns with the findings by Cheng, Huang [38] on promoting CW utilization, which highlight that combining fiscal incentives, technical services, and long-term procurement contracts at the design stage can generate cost advantages and produce replicable business cases. Finally, technological progress (O3)—including mobile crushing equipment, online testing, and digital monitoring—was emphasized by respondents as a “double-edged sword” capable of simultaneously addressing both cost and enforcement challenges (P5, P6, P7). Policy design should therefore prioritize the establishment of technology demonstration funds and standardized technology access requirements, making the adoption of advanced technologies a precondition for receiving subsidies or qualifying for demonstration projects [39]. Furthermore, recent empirical work has demonstrated that green technology adoption and digital transformation play critical roles in waste management innovation and carbon reduction across major economies [40]. Such an approach would achieve a dual outcome: enhancing supply-side efficiency while strengthening regulatory capacity.

(4) Discussion on the threat

Illegal dumping and the difficulty of law enforcement and evidence collection (T1) emerged as one of the most pressing concerns among government respondents. Evidence indicates that, in certain areas, the overall cost of illegal disposal remains lower than that of compliant practices, placing law-abiding enterprises at a competitive disadvantage. At the same time, insufficient demand for recycled materials and their weak price competitiveness (T2), together with the time-limited and unstable nature of fiscal subsidies (T3), were strongly emphasized by frontline practitioners. Respondents noted that T2 directly constrains long-term investment decisions by enterprises, and argued that short-term procurement subsidies are needed to offset market price gaps during the transition period. However, in addressing T3, policy designers should consider measures beyond direct fiscal support. For example, introducing diversified financing mechanisms-such as green finance, public-private-partnership (PPP) models, and performance-based contracting-could reduce overreliance on local annual budgets [41].

### 3.3. Delphi validation results

Based on the preliminary interview findings in Section 3.2 (candidate SWOT elements), this study simulated a two-round Delphi evaluation with four experts specializing in policy research in Xi’an. In each round, the experts rated the importance of each candidate element on a five-point scale (1–5). During the first round, they also provided written comments. The research team then synthesized the feedback and returned aggregated statistical results to the experts for reconsideration in the second round. To determine prioritization, the mean scores from the second round were normalized to generate the final weights. The results are presented in **Table 5**.

**Table 5:** Final SWOT elements with round 2 mean scores and normalized weights.

Element	Expert Scores (1–5)	Mean (R2)	SD	Normalized Weight (Weight = Mean/ ΣMean)
S1	4, 4, 5, 4	4.2	0.50	0.0886
S2	3, 4, 3, 3	3.4	0.48	0.0717
S3	3, 3, 4, 3	3.2	0.50	0.0675
W1	5, 4, 5, 5	4.8	0.50	0.1013
W2	4, 4, 4, 4	4.0	0.00	0.0844
W3	4, 3, 4, 4	3.8	0.48	0.0802
O1	4, 5, 4, 4	4.2	0.50	0.0886
O2	4, 4, 5, 4	4.2	0.50	0.0886
O3	3, 4, 3, 4	3.4	0.48	0.0717
T1	5, 4, 5, 5	4.8	0.50	0.1013
T2	3, 3, 4, 3	3.4	0.48	0.0717
T3	4, 4, 4, 4	4.0	0.00	0.0844
**Total**		**47.4**		**1**

As shown in Table 5, the mean values range from 3.2 to 4.8, while standard deviations (SD) values mostly remain below 0.6, indicating a high level of agreement among experts. Following the approach recommended by Okoli and Pawlowski, items with SD < 0.8 were retained as validated core factors [42]. Overall, experts assigned the highest importance to “delays in subsidy disbursement and verification (W1)” and “illegal dumping and difficulties in enforcement (T1),” both with mean scores of 4.8 and normalized weights of approximately 0.101. This indicates a strong consensus that execution efficiency and enforcement/compliance issues represent the two most urgent bottlenecks in Xi’an’s CDM. The next tier of priorities included “limited testing capacity and high compliance costs (W2),” “green finance opportunities and urban renewal pilots (O1, O2),” and “fiscal instability of subsidies (T3).” This distribution reflects that, in addition to implementation efficiency, the expert panel also emphasized the need to combine short-term fiscal incentives with long-term market-oriented instruments and capacity-building in testing and technology. Subsequent TOWS matrix analysis and strategy prioritization will therefore focus on these high-weighted elements as the core starting points.

### 3.4. TOWS-based strategic recommendations

**Fig 2:**
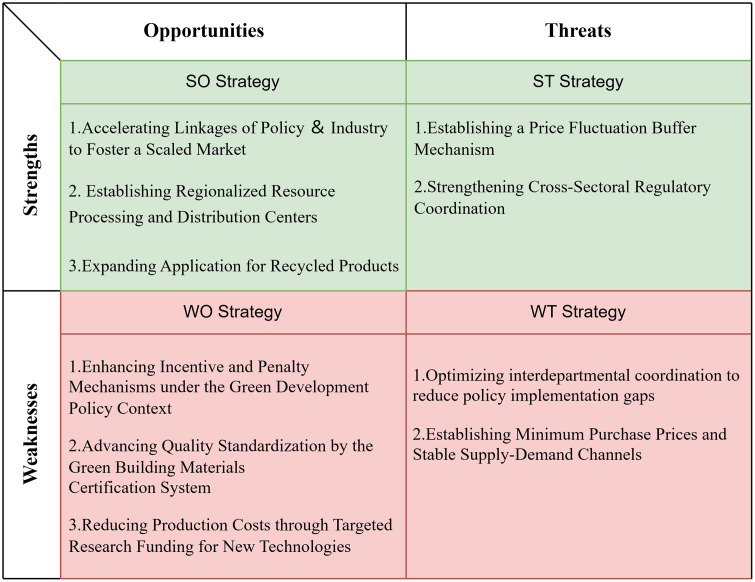
Recommendations for policy improvement at TOWS basis.

Building on the preceding SWOT analysis and the Delphi validation results, this study systematically matches the strengths, weaknesses, external opportunities, and external threats of Xi’an’s CWM policies to construct a TOWS matrix and generate targeted policy recommendations. The proposed strategies are organized into four categories: SO (leveraging strengths to seize opportunities), ST (leveraging strengths to mitigate threats), WO (addressing weaknesses by capitalizing on opportunities), and WT (minimizing weaknesses to counteract threats). As summarized in Fig 2, these four categories of strategies are designed not only to enhance policy implementation and market mechanisms but also to balance the interests of government authorities, construction enterprises, and recycling firms. Ultimately, the aim is to foster long-term progress in the reduction, resource utilization, and safe disposal of CW.

#### 3.4.1. SO-leveraging strengths to seize opportunities.

**Accelerating linkages of policy and industry to foster a scaled market:** The Regulations on the Resource Utilization of CW in Xi’an (2022) explicitly stipulate requirements for disposal procedures, product standards, and market applications, reflecting the local government’s active response to the green and low-carbon development agenda. In previous studies on CW, the resource utilization rate of waste was often regarded as an indicator of the effectiveness of local CWM. The proportion of recycled aggregates, sand, bricks/blocks, regenerated building materials, and other secondary materials that re-enter the construction supply chain is defined as waste resource utilization rate [43]. According to statistics from the Xi’an Municipal Housing and Urban-Rural Development Bureau, the city generated approximately 103.96 million tons of CW in 2024, with a resource utilization rate nearly 50%. By referring to the same economic level cities like Haikou and Qingdao, their utilization rate of CW is 73.8% and 83.2% in 2024 respectively [44,45].

Interviews with recycling enterprises revealed a common concern: although policy frameworks are in place, the absence of binding mechanisms linking resource utilization to engineering projects has weakened the competitiveness of recycled products in the market. Zhang, Shi [46] suggested that the government could establish mandatory usage ratios (e.g., requiring minimum percentages of recycled aggregates in road construction or public housing projects) and incorporate such criteria into bidding processes, thereby creating stable demand. Prior research also highlights that a dual policy-market driving mechanism is essential for scaling the resource utilization industry, and that coupling recycled products with engineering procurement is an effective way to overcome market bottlenecks [47]. Such measures would not only expand market capacity but also attract private investment and technological innovation, generating a positive feedback loop across the industrial chain.

**Establishing regionalized resource processing and distribution centers:** Field investigations indicate that most CW treatment facilities in Xi’an are currently located in peripheral suburban areas, with average one-way transport distances often exceeding 20 km. This not only increases fuel consumption and disposal costs but also discourages enterprises from actively participating in resource recycling. Considering the spatial distribution of Xi’an’s industrial parks and logistics hubs--such as the International Port District, Jingwei New City, and Lintong Industrial Zone, the city has favorable conditions for developing distributed processing and distribution centers. Such centers could be equipped with integrated crushing, screening, washing, and grading facilities to enable proximate treatment and secondary processing, thereby reducing transport distances and improving overall cost efficiency. Comparable practices have been adopted in other contexts, for example, Tokyo has established multiple small-scale recycled aggregate plants across its 23 special wards, achieving an annual recycling rate above 90% while reducing carbon emissions through localized consumption [48]. Theoretically, this approach aligns with the “local loop principle” of circular economy, which emphasizes minimizing resource transfer distances and strengthening urban resource closed-loop systems [49]. For Xi’an, the construction of regionalized centers would not only alleviate treatment bottlenecks but also form a stable supply chain to support downstream market expansion.

**Expanding application for recycled products:** Xi’an is currently experiencing a peak period of urban renewal and large-scale infrastructure development. During the 14th Five-Year Plan, the city has scheduled the construction or renovation of seven metro lines and an additional 24.46 million square meters of building space, providing substantial potential demand scenarios for recycled products [50]. Interviews with recycling enterprises revealed that although low-end backfill materials account for large shipment volumes, they suffer from low profit margins and intense competition. In contrast, higher value-added products such as permeable bricks, prefabricated components, and landscaping materials are more readily accepted by design institutes and government projects, while also aligning with the policy agenda of green buildings and sponge city development. Previous studies emphasize that product diversification and the establishment of standardized testing systems are key to enhancing market acceptance of recycled materials [51]. Experiences from cities such as Guangzhou and Nanjing demonstrate that providing research subsidies for CW-derived aggregates in municipal landscape and sponge city projects can significantly increase both industrial value-added and application scope [52]. Drawing on these practices, Xi’an could channel policy funding toward technological research and foster industry collaboration to develop local standards. By encouraging design institutes and contractors to integrate recycled materials at the early stages of project planning, the city can drive a transition from “low-end disposal” to “high-value utilization.”

#### 3.4.2. ST-leveraging strengths to address threats.

**Establishing a price fluctuation buffer mechanism:** Interviews with recycling enterprises revealed a recurring concern: when the price of natural aggregates drops, demand for recycled aggregates declines sharply, directly eroding profit margins and, in some cases, forcing production lines into idleness. Although Xi’an has issued the Administrative Measures for the Resource Utilization of CW, it currently lacks concrete instruments to stabilize recycled product markets during such fluctuations. Drawing on experiences like price-differential compensation mechanism, the government could establish a dedicated fund to dynamically monitor and offset price gaps between natural and recycled aggregates [53]. This approach would safeguard the price competitiveness of recycled products, thereby strengthening confidence among both recycling operators and construction enterprises while reducing the risk of industry contraction caused by short-term shocks. Moreover, as Xi’an promotes its green and low-carbon construction strategy, such a mechanism could be linked to carbon-reduction accounting, whereby energy-saving and emission-reduction performance directly influence the degree of compensation, thus reinforcing a virtuous cycle of policy incentives and market stability.

**Strengthening cross-sectoral regulatory coordination:** Respondents from both construction and recycling firms highlighted illegal dumping and the circulation of low-quality recycled materials as major threats to the healthy development of the industry in Xi’an. While the city has established a comprehensive urban management system involving multiple enforcement agencies, regulatory responsibilities often overlap or leave gaps--particularly among the Housing and Urban-Rural Development Bureau, Environmental Protection Bureau, and Urban Management Bureau-diminishing enforcement effectiveness. Lessons show that real-time information sharing and joint enforcement across government agencies can effectively curb illegal dumping and prevent malpractice in the recycled materials market [54]. For Xi’an, the existing “Smart Urban Management” platform offers a technical foundation. By integrating transportation monitoring, project registration, enterprise licensing, and product sales flows into a unified regulatory database, the city could enhance backend monitoring and increase the frequency of product inspections.

#### 3.4.3. WO strategies: Seizing opportunities to overcome weaknesses.

**Enhancing incentive and penalty mechanisms under the green development policy context:** Since 2022, Xi’an has issued several policy frameworks, including the 14th Five-Year Comprehensive Energy Conservation and Emission Reduction Plan and the Green Building Materials Promotion Action Plan. However, interviews with recycling enterprises suggest that the current incentive measures remain insufficiently detailed and lack targeted support for the production and application of recycled aggregates. As one respondent noted, “The reward standards do not differentiate by production scale or energy-saving and emission-reduction performance, so the incentive effect is limited.” Existing research demonstrates that performance-based, tiered reward mechanisms can effectively enhance enterprise motivation in green transitions [55]. Furthermore, setting differentiated subsidies based on factors such as the proportion of recycled materials used, product quality compliance rates, and carbon-reduction outcomes, while imposing strict penalties on long-term noncompliance and illegal dumping can significantly strengthen policy implementation and provide clearer guidance for industry behavior [56]. At same time, comparative analyses across the BRICS economies reveal that sustainability of environmental management depends not only on financial inputs but also their efficiency [57]. For Xi’an, refining incentive and penalty indicators within the current policy framework would not only increase policy effectiveness but also encourage enterprises to actively improve their technological processes, thereby raising the overall performance of the sector.

**Advancing quality standardization through the green building materials certification system:** Interview results reveal that construction enterprises remain cautious about the long-term performance and durability of recycled aggregate products, often adopting a wait-and-see attitude in procurement decisions. Since Xi’an was designated in 2018 as a national pilot city for CWM, the city has also piloted certification schemes for green building materials. However, certification frameworks covering recycled aggregates remain underdeveloped. Su, Si [58] argues that a unified and enforceable standard for recycled products is essential to improving market acceptance. For Xi’an, this could involve integrating national green building material certification with locally adapted standards to develop more detailed performance indicators and testing protocols. In parallel, accredited third-party testing platforms could be introduced to provide transparent and publicly accessible product performance data [59]. Such measures would simultaneously reduce market hesitation and foster a competitive environment in which higher-quality products prevail, thereby driving industry-wide improvement.

**Reducing production costs through targeted research funding for new technologies:** At present, both construction enterprises and recycling operators report that energy consumption and labor expenses in the processes of crushing, sorting, and impurity removal account for a substantial share of total costs. As one recycling enterprise manager observed, “*our sorting costs are rising; to keep the company alive, product prices inevitably increase, which in turn reduces our market share*.” This somehow reflects that the local recycled aggregate industry remains in a cost-sensitive stage of development. Although the Xi’an Municipal Bureau of Science and Technology and the Development and Reform Commission have prioritized green and low-carbon technological research in recent years, support specifically for CW recycling technologies has been limited. In Japan, substantial public investment has been directed toward research in waste recycling technologies, fostering collaboration between enterprises, universities, and research institutes. These efforts have yielded significant advances in crushing equipment, intelligent sorting systems, and other technological innovations, with pilot projects confirming both technical and economic feasibility [60]. For Xi’an, a similar approach could be pursued by reallocating or expanding existing green technology funding streams, with a clear preference for recycling-related equipment and process innovation. By lowering the cost differential with natural aggregates, such initiatives would provide the industry with sustainable pathways for technological upgrading and long-term competitiveness.

#### 3.4.4. WT-minimizing weaknesses to address threats.

**Optimizing interdepartmental coordination to reduce policy implementation gaps:** In addition to the regulatory blind spots among government agencies discussed earlier, interviewees from recycling enterprises also reported problems such as delayed information transmission across departments and cumbersome approval procedures, which significantly hinder the timely initiation of projects and the disbursement of subsidies. These obstacles substantially weaken firms’ incentives to engage in recycling. As Hackel, Schönhals [61] argues, the establishment of a digitalized project ledger can enable end-to-end tracking from project initiation and approval to funding allocation, thereby preventing policy failure caused by administrative barriers. Building on this insight, Xi’an could adopt a “multi-plan integration” approval platform, incorporating green development policies into a unified coordination mechanism to minimize the time lag between policy issuance and actual implementation.

**Establishing minimum purchase prices and stable supply-demand channels:** Interviews with recycling enterprises also revealed challenges they share with transportation partners, namely unstable demand for CW and significant fluctuations in recycling prices. Although Xi’an has piloted government-led centralized recycling systems in certain districts, measures to mitigate market price volatility remain absent, and no unified minimum purchase price mechanism has been introduced. Research on market stabilization indicates that establishing guaranteed purchase schemes or minimum price mechanisms is an effective measure to enhance the sector’s resilience to external shocks [62]. For example, the local government introduced long-term procurement contracts and a unified settlement platform that integrates construction firms, recycling enterprises, and key project owners. This system conducts risk assessments at critical nodes of the supply chain, thereby reducing the likelihood of chain disruptions during market downturns [63]. Drawing on this experience, implementing a minimum purchase price mechanism in Xi’an would help secure a stable supply of raw materials for recycled aggregate producers and safeguard the basic revenues of recycling enterprises.

### 3.5. Policy implications

#### 3.5.1. Comparison with other chinese cities.

Compared with the well-managed cities such as Shenzhen and Suzhou, Xi’an’s CWM policies show a more localized, incentive-driven approach rather than a purely regulatory one. While Shenzhen emphasizes marketization and digital traceability, Xi’an’s experience demonstrates that financial incentives and urban renewal pilots can mobilize participation even under limited fiscal capacity [5]. Unlike Suzhou, where industrial symbiosis has already formed mature recycling networks, Xi’an’s emerging model relies heavily on policy-led collaboration between contractors and recyclers, providing valuable insights for other developing cities in western China [21].

#### 3.5.2. Transferability and lessons learned.

Findings from the SWOT–Delphi analysis suggest that Xi’an’s experience can inform medium-sized inland cities facing similar constraints. Specifically, the integration of municipal subsidies with urban renewal projects planning (S1, O2) could be replicated in urban renewal programs nationwide. The city’s cross-departmental challenges (W3) also highlight the importance of digital information platforms for data sharing-an area that could be institutionalized at the provincial level. Furthermore, promoting green finance instruments (O1) and ensuring long-term policy stability (T3) are not only local needs but also critical to national circular economy promotion.

#### 3.5.3. Relevance to national and international agendas.

At a broader policy level, Xi’an’s experience resonates with the China’s 14th Five-Year Plan for Circular Economy Development, and the One Belt One Road (OBOR) strategy, which emphasizes sustainable infrastructure and regional green cooperation [64,65]. As Xi’an is a key node city in the OBOR corridor, its CWM framework demonstrates how local governments can balance economic development and environmental governance through policy-driven innovation. The city’s practices also offer lessons for other developing economies involved in OBOR on how localized incentives and multi-stakeholder engagement can enhance the sustainability of urban construction.

## 4. Conclusion

Taking Xi’an as a case study, this research employed policy document analysis, semi-structured interviews, and the Delphi method to systematically examine the incentive mechanisms embedded in CDWM policies. The SWOT results reveal that Xi’an possesses notable strengths in fiscal support and the establishment of standards, but also faces weaknesses such as low subsidy disbursement efficiency and insufficient interdepartmental coordination. Externally, the green development agenda and emerging technologies create new opportunities, yet low market acceptance, enforcement challenges, and fiscal uncertainty remain prominent threats. Building on the TOWS framework, this study proposes optimization strategies across short-, medium-, and long-term horizons: in the short term, closing regulatory loops and enhancing enforcement; in the medium term, strengthening capacity building and market confidence; and in the long term, institutionalizing and scaling up development to enable a transition from a “policy-driven” to a “market-driven” model.

By combining policy interpretation with stakeholder interviews and applying a SWOT-TOWS analytical process, this study provides targeted recommendations for improving CDWM policies in Xi’an, while also suggesting a phased pathway—first strengthening enforcement, then building capacity, and ultimately advancing marketization—that may hold broader relevance for similar cities. Nonetheless, limitations should be noted: the findings are based on a single-city case with a limited sample of interviewees and experts, and rely primarily on qualitative analysis. Consequently, the conclusions are highly context-specific, and caution is required when generalizing or transferring the insights to other regions. Future research should expand to larger samples and cross-city comparisons, incorporate quantitative assessments, and conduct pilot evaluations with cost-benefit analyses of core interventions (such as electronic subsidy disbursement, shared testing centers, and minimum purchase price mechanisms). Empirical evidence from such studies would help verify the scalability and long-term effectiveness of the strategies proposed.

## Appendix A. Interview questions

I Effectiveness or positive impacts

The first set of questions primarily aims to explore the effectiveness or positive impacts of Xi’an’s existing policies in practical operation. Interviewees will elaborate through the following questions:

1. In which aspects do you believe the current incentive policies for Construction and Demolition Waste (C&DW) management in Xi’an are most effective?
2. Have these policies played a positive driving role in your work/management practices? Are there any successful cases you could share?
3. Compared to previous policies, have you perceived a significant improvement in the enforcement capability or resource support of the current policies?

II The problems and difficulties

The second set of questions primarily aims to identify the problems and difficulties inherent in the policies themselves or encountered during their implementation. Interviewees will elaborate through the following questions:

1. Based on your practical experience or execution process, what shortcomings or operational obstacles do you think exist in these incentive policies?
2. During the implementation process, are there issues such as difficulties in execution, unclear incentives, or a mismatch with the actual situation?
3. Have you encountered situations where policies failed to achieve their intended effectiveness due to imperfect institutional design or poor inter-departmental coordination?

III The external environment or emerging trends

The third set of questions primarily aims to understand the external environment or emerging trends that are currently or potentially favorable for policy improvement and implementation. Interviewees will elaborate through the following questions:

1. What opportunities in current technological, market, or regulatory trends do you believe could be leveraged to further optimize the policy incentive mechanisms?
2. Against the backdrop of rising public environmental awareness and green development policies, are there new cooperation opportunities or development directions emerging?
3. Are there new materials, equipment, or platforms that could facilitate the sorting, recycling, and resource recovery of C&DW, thereby better supporting policy implementation?

IV The external environment

The fourth set of questions primarily aims to investigate factors in the external environment that may hinder policy implementation and effectiveness. Interviewees will elaborate through the following questions:

1. In the current execution of C&DW management policies, are there external threats from sources such as industry inertia, conflicting interests, or regulatory gaps?
2. In your opinion, what is the most significant challenge facing the current policies? Does it stem from the policies themselves, or from a lack of cooperation from the external environment?
3. Are there any legal, institutional, or market mechanism limitations that prevent the incentive policies from achieving their expected outcomes?
